# Macular microvascular parameters in the ganglion cell-inner plexiform layer derived by optical coherence tomography angiography: Vascular structure-central visual function analysis

**DOI:** 10.1371/journal.pone.0240111

**Published:** 2020-10-01

**Authors:** Cody Hansen, Karine D. Bojikian, Zhongdi Chu, Xiao Zhou, Qinqin Zhang, Raghu C. Mudumbai, Murray A. Johnstone, Ruikang K. Wang, Philip P. Chen

**Affiliations:** 1 University of Washington School of Medicine, Seattle, WA, United States of America; 2 Department of Ophthalmology, University of Washington, Seattle, WA, United States of America; 3 Department of Bioengineering, University of Washington, Seattle, WA, United States of America; Bascom Palmer Eye Institute, UNITED STATES

## Abstract

**Purpose:**

To investigate the relationships between global and sectoral macular vascular microcirculation parameters in the ganglion cell-inner plexiform layer (GCIPL) assessed by optical coherence tomography angiography (OCTA), and global and sectoral visual field (VF) central mean sensitivity (CMS) assessed by standard automated perimetry.

**Methods:**

Fifty-four eyes with open angle glaucoma were scanned using a swept-source OCTA (Plex Elite 9000, Zeiss, Dublin, CA) and macular vascular microcirculation was measured by calculating the overall flux and vessel area density (VAD) over the entire 6mm x 6mm area, excluding large retinal vessels. Central 10-degree VF CMS was calculated based on 24–2 VF. Pearson correlation was used to investigate the correlation between global and sectoral OCTA parameters and global and sectoral VF CMS.

**Results:**

Both global GCIPL flux and VAD were significantly correlated with VF CMS (p<0.001). For the sectoral analysis, sectoral VAD was significantly correlated with sectoral VF CMS in all comparisons except for the inferonasal VF CMS with supero-temporal (ST) GCIPL VAD (p = 0.097). Although highest correlation was observed for both ST VF CMS with inferior GCIPL VAD and infero-temporal VF CMS with superior GCIPL VAD (r = 0.683, p<0.001), there was no significant difference in correlation when compared to the global VAD and other sectors' correlation coefficients (p≥ 0.091), except for the ST GCIPL VAD (p = 0.001).

**Conclusions:**

Global and sectoral macular vascular microcirculation in the GCIPL, as determined by OCTA, was significantly correlated with global and sectoral VF CMS in glaucomatous patients. OCTA can aid in the understanding of the structure-function relationships of the macular region.

## Introduction

Glaucoma is a leading cause of irreversible blindness and diagnosis is frequently delayed due to asymptomatic disease progression until relatively late stages [1]. Projections show that 79.6 million people will be diagnosed with glaucoma by 2020, with 11.2 million suffering from bilateral blindness [2]. Glaucoma has many different subtypes but defines a group of progressive optic neuropathies characterized by degeneration of retinal ganglion cells (RGCs) and changes in the optic nerve, retinal nerve fiber layer (RNFL), and associated visual field (VF) defects [1]. About 50% of retinal ganglion cells lie in the macular region [3]. This retinal nerve fiber layer of RGCs has been shown to be damaged in most cases of glaucoma and correlates with VF defects even in early stage disease [4]. One study using High Definition Optic Coherence Tomography (HD-OCT) showed that the association between average macular ganglion cell inner plexiform layer (GCIPL) thickness and the central cluster VF sensitivity was significantly stronger than that of temporal peripapillary RNFL thickness (p < 0.001) [5].

Among many risk factors for the development of glaucoma, only intraocular pressure (IOP) has been found to be modifiable [6]. Other risk factors have been described that may affect ocular blood flow, including hypertension, diabetes, smoking, and vasospasm [7]. Imaging and measurements of blood flow have provided insight into exploring these blood flow related risk factors and how they affect overall ocular blood flow and function. A recent imaging modality, optical coherence tomography angiography (OCTA), can generate a three-dimensional (3D) image of both blood flow and structural information in the retina and choroid without the prior injection of dye. Earlier studies using OCTA-collected data demonstrated that blood flow measurements of the optic disc, peripapillary retina, and macula are associated with the severity of VF defects in glaucomatous patients and are significantly different when compared to normal eyes [8–17].

Automated static perimetry is the standard test used for assessing peripheral retinal sensitivity in patients with glaucoma. The purpose of the present study is to investigate the relationships between global and sectoral VF central (10 degree) mean sensitivity (CMS), and global and sectoral macular vascular microcirculation parameters in the ganglion cell-inner plexiform layer. We hypothesized that global and sectoral macular vascular microcirculation detected by OCTA correlates with disease severity and corresponding sectoral VF central mean sensitivity measurements in glaucomatous eyes and sought to evaluate whether any sector has more significant correlation compared to global or other sectors correlations.

## Methods

### Subjects

This study was approved by the Institutional Review Board of the University of Washington (UW) and informed consent was obtained from all subjects before imaging. This study followed the tenets of the Declaration of Helsinki and was conducted in compliance with the Health Insurance Portability and Accountability Act.

Patients with diagnosis of open-angle glaucoma were prospectively enrolled at the UW Medicine Eye Institute. Inclusion criteria were defined as best-corrected visual acuity of 20/40 or better and refractive error between -6.0 and +3.0 D spherical equivalent. Exclusion criteria were defined as significant media opacity preventing high-quality imaging, any ocular disease other than glaucoma or cataract, and previous intraocular surgeries other than uncomplicated glaucoma or cataract surgery. Diagnosis of open-angle glaucoma was based on (1) optic disc rim defect (thinning or notching) or RNFL defect visible through either slit-lamp biomicroscopy or optic coherence tomography (OCT) scan; and (2) glaucomatous VF loss. Each subject received a comprehensive ophthalmologic exam and underwent a VF exam to determine mean deviation (MD) and pattern standard deviation (PSD). All VFs were performed on a Humphrey Field Analyzer II (Carl Zeiss Meditec, Dublin, CA), and only reliable tests were included (<20% fixation loss and <15% false positive response rates). Patients were classified into stages based on HVF MD value: mild stage has MD no worse than 6.00 dB; moderate stage has MD worse than -6.00 dB but no worse than -12.00 dB; severe stage has MD worse than -12.00 dB. Visual field central mean sensitivity (1/Lambert, L) was calculated by averaging the anti-log absolute sensitivity values within the central 10 degrees (12 tested points) area [18]. One eye from each subject was included in this study. A single eye was selected based on image quality if both were eligible.

Blood pressure (BP) was measured in a seated position using the Welch Allyn (Model LXI #4700–60; Welch Allyn, Skaneateles Falls, New York) automatic BP monitor. The BP was measured once at the same visit immediately after the OCTA scan to calculate mean ocular perfusion pressure (MOPP). MOPP was defined as 2/3 (mean arterial pressure—IOP), where mean arterial pressure = diastolic BP + 1/3(systolic BP–diastolic BP).

### Image acquisition and scanning protocol

All subjects were scanned centered at the foveola using swept-source OCTA (Plex Elite 9000, Zeiss, Dublin, CA), characterized by a central wavelength of 1050 nm, a bandwidth of 100 nm, and a 100 kHz scanning rate. Each scan consisted of 500 A-scans within one B-scan and 500 B-scan clusters (2 repeats at each transverse location) covering a 6 mm x 6 mm scanning area. The scanning depth was 3.0 mm in tissue with 1536 sampling pixels. Blood flow signals were extracted using a complex optical microangiography (OMAG) method and exported from the Plex Elite device. A semi-automatic retinal layer segmentation program was applied to the structural OCT images to precisely separate the GCIPL from the outer boundary of the nerve fiber layer to the outer boundary of the inner plexiform layer [19]. Macular vascular *en face* images were generated using maximum projection. Macular vascular microcirculation was then measured as previously described by calculating the overall flux, vessel area density (VAD), vessel diameter index (VDI), vessel skeleton density (VSD), vessel perimeter index (VPI) and vessel complexity index (VCI) over the entire 6mm x 6mm area excluding large retinal vessels [20]. Measurements of GCIPL microvasculature (VAD, VDI, VSD, VPI, and VCI) measure approximately the same thing (i.e. vessel density), but there are slight differences. Vessel area density calculates all the areas occupied by the vessels within the scanned area (i.e. vessel density) while VSD provides vessel length information. Vessel skeleton density has a stronger emphasis on capillaries as it counts only the vessel length, not diameter. Vessel diameter index calculates the averaged vessel diameter, as calculated by VAD/VSD (area/length). Vessel perimeter index is the density of vessel perimeters. This parameter provides little extra information beyond VAD and VSD. Vessel complexity index calculates the morphological complexity and, therefore, provides vessel branching information beyond VAD and VSD, similar to the information provided by fractal dimension. The method for large retinal vessel removal has been described previously [8]. In brief, a multiscale Hessian filter was developed to detect blood vessels with various diameters, and large vessels of more than 32 μm were removed. Next, the sectoral (supero-temporal, superior, supero-nasal, infero-nasal, inferior, infero-temporal) blood flow metrics were measured in an ‘elliptical annulus (dimensions, vertical inner and outer radius of 0.5 mm and 2.0 mm, horizontal inner and outer radius of 0.6 mm and 2.4 mm, respectively)’ [5], therefore, parts of the 6x6 scans were not included in the analysis. Scans with an OCT signal strength less than 7 were excluded from analysis (as recommended by the manufacturer).

Sample size was calculated using G*Power 3.1 [21]; with p significant at 0.05 and 90% power, a total sample size of 38 was estimated to achieve 0.5 correlation between two variables.

### Statistical analysis

**Fig 1:**
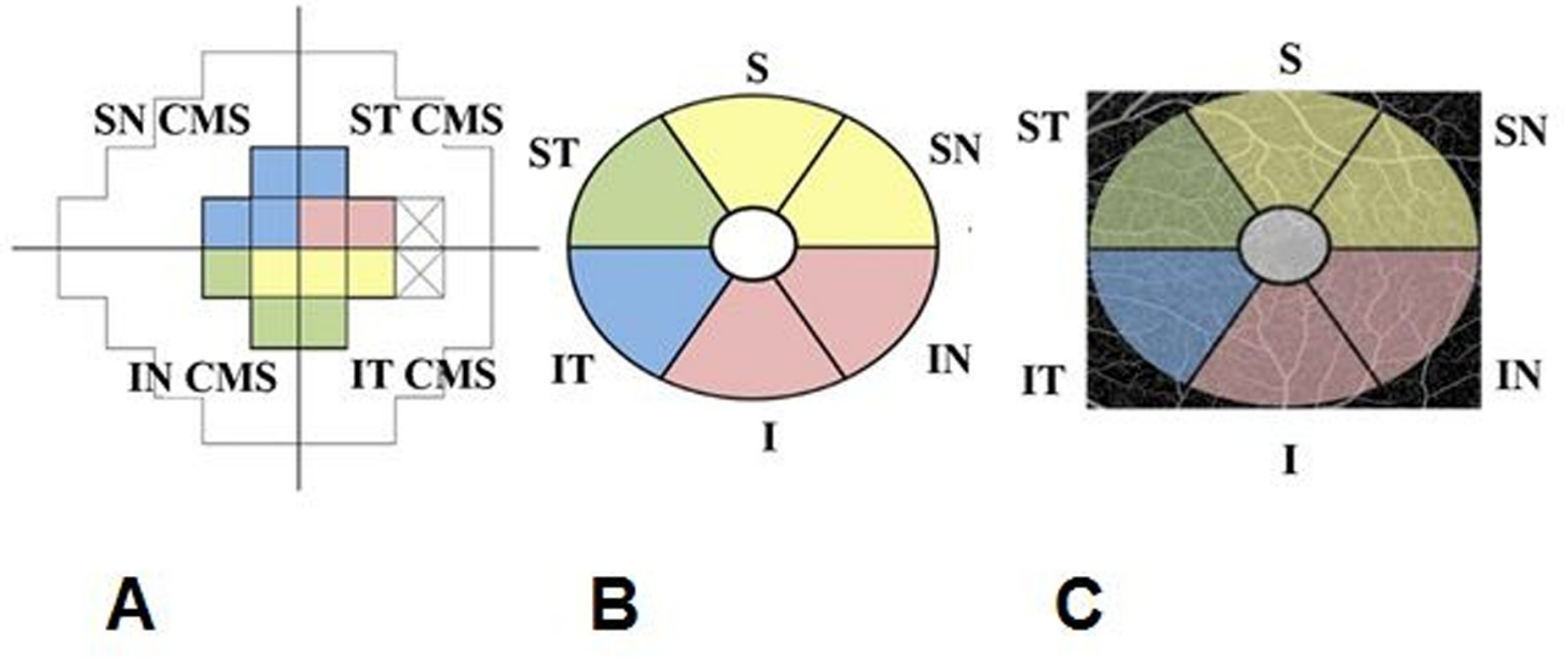
Representation of cluster of central 12 points on 24–2 Humphrey visual field (CMS–central mean sensitivity) (A) and corresponding sectors on macular scans (B, C). SN = super-nasal, IN-infero-nasal, ST = supero-temporal, IT = infero-temporal, I = inferior, S = superior.

Pearson correlation was used to investigate the correlation between global and sectoral OCTA parameters and global and sectoral VF CMS adapted from the structure-function correspondence map suggested by Garway-Heath et al. [22]. The supero-nasal (SN) VF CMS was defined as the average VF CMS in 4 supero-nasal points of the 12 central cluster points and the infero-nasal (IN) VF CMS was defined as the average VF CMS in the 3 inferonasal points. The supero-temporal (ST) VF CMS was defined as the average VF CMS in the 2 supero-temporal points and the infero-temporal (IT) VF CMS was defined as the average VF CMS in 3 infero-temporal points (Fig 1). Fisher’s r to z transformation was used to compare the correlation coefficients. Pearson correlation and locally weighted scatterplot smoothing (LOWESS) curves were also used to fit the relationship graphically. A p-value of p < 0.05 was considered statistically significant.

## Results

We included fifty-four eyes from 54 enrolled subjects diagnosed with open-angle glaucoma (Table 1). Baseline characteristics are shown in Table 1. The average VF MD, VF PSD, and VF CMS of these subjects was -7.9 ± 7.6 dB, 6.9 ± 4.3 dB, and 612.8 ± 307.3 (1/L), respectively.

**Table 1:** Baseline information and optical coherence tomography angiography findings in the macular ganglion cell inner plexiform layer among glaucoma subjects (N = 54).

	Glaucoma (N = 54)
Age (y)	69.4 ± 12.3
Male / Female	33 (61.1%) / 21 (39.9%)
Systolic Blood Pressure (mmHg)	127.7 ± 16.8
Diastolic Blood Pressure (mmHg)	78.3 ± 9.2
MOPP (mmHg)	52.3 ± 8.0
Systemic Hypertension, n (%)	23 (42.6%)
Diabetes Mellitus, n (%)	5 (9.3%)
Systemic Hypertension Medications, Yes	22 (40.7%)
Glaucoma Severity	
Mild	27 (50.0%)
Moderate	14 (25.9%)
Severe	13 (24.1%)
Intraocular Pressure (mmHg)	12.7 ± 3.3
Number Glaucoma Medications	2.0 ± 1.2
Central Corneal Thickness μ	536.2 ± 34.6
History of Glaucoma Surgery, Yes	11 (20.4%)
Cup-to-disc Ratio	0.78 ± 0.15
VF MD (dB)	-7.9 ± 7.6
VF PSD (dB)	6.9 ± 4.3
VF CMS Parafoveal Area (1/L)	612.8 ± 307.3
VF CMS SN Area (1/L)	554.9 ± 374.6
VF CMS IN Area (1/L)	665.5 ± 336.0
VF CMS ST Area (1/L)	649.4 ± 372.4
VF CMS IT Area (1/L)	777.4 ± 298.7
RNFL Thickness (μm)	61.6 ± 14.1
Global GCIPL Flux	0.255 ± 0.029
Global GCIPL	0.480 ± 0.048
Vessel Area Density	
Global GCIPL Vessel Diameter	13.598 ± 0.253
Global GCIPL	
Vessel Skeleton Density	0.246 ± 0.023
Global GCIPL Vessel Perimeter Index	0.450 ± 0.043
Global GCIPL Vessel Complexity Index	6215.59 ± 573.06

MOPP = mean ocular perfusion pressure, RNFL = retinal nerve fiver layer; VF = visual field; MD = mean deviation, PSD = pattern standard deviation, CMS = central mean sensitivity GCIPL = ganglion cell inner plexiform layer.

Global GCIPL thickness and global OCTA parameters were significantly correlated to functional and structural clinical measurements, except for global GCIPL thickness and VF PSD (p = 0.109) (Table 2).

**Table 2:** Pearson correlation results between global GCIPL thickness and global OCTA parameters, and other functional and structural clinical measurements (N = 54).

	Global GCIPL Thickness (μm) Correlation (r)	*P-value*	Global GCIPL Flux	*P-value*	Global GCIPL VAD	*P-value*
			Correlation (r)		Correlation (r)	
GCIPL Thickness (μm)			0.526	**<0.0001**	0.228	0.094
Cup-to-disc Ratio	-0.349	**0.009**	-0.532	**<0.0001**	-0.586	**<0.0001**
VF MD (dB)	0.390	**0.003**	0.624	**<0.0001**	0.623	**<0.0001**
VF PSD (dB)	-0.221	0.109	-0.486	**<0.0001**	-0.516	**<0.0001**
Global VF CMS parafoveal area (1/L)	0.272	**0.047**	0.629	**<0.0001**	0.718	**<0.0001**

VF = visual field, MD = mean deviation, PSD = pattern standard deviation, CMS = central mean sensitivity GCIPL = ganglion cell inner plexiform layer, VAD = vessel area density.

All global GCIPL OCTA parameters (flux, VAD, VSD, VPI, and VCI) were significantly correlated with VF CMS (p<0.001), except for global GCIPL vessel diameter and global VF CMS (p = 0.251) (Table 3).

**Table 3:** Pearson correlation between global OCTA parameters global visual field sensitivities for the glaucoma patients (N = 54).

	Correlation (r =)	*P-*value
Global GCIPL flux and Global VF CMS	0.629	**<0.001**
Global GCIPL vessel area density and Global VF CMS	0.718	**<0.001**
Global GCIPL vessel diameter and Global VF CMS	0.159	0.251
Global GCIPL vessel skeleton density and Global VF CMS	0.713	**<0.001**
Global GCIPL vessel perimeter index and Global VF CMS	0.708	**<0.001**
Global GCIPL vessel complexity index and Global VF CMS	0.691	**<0.001**

VF = visual field, CMS = central mean sensitivity GCIPL = ganglion cell inner plexiform layer.

For the sectoral analysis, sectoral VAD was significantly correlated with sectoral VF CMS in all comparisons, except for the ST GCIPL VAD and IN VF CMS (p = 0.097) (Table 4). Although inferior (I) GCIPL VAD and superior (S) GCIPL VAD showed the highest correlation with their correspondent VF CMS (r = 0.683, p<0.001), there was no significant difference when compared to the global VAD and other sectors correlation coefficients (p≥ 0.091), except for the ST GCIPL VAD (p = 0.001).

**Table 4:** Pearson correlation between sectoral OCTA parameters and sectoral visual field sensitivities for the glaucoma patients (N = 54).

	Correlation (r =)	*P-*value
IT GCIPL vessel area density and SN VF CMS	0.559	**<0.001**
ST GCIPL vessel area density and IN VF CMS	0.228	0.097
IN GCIPL vessel area density and ST VF CMS	0.539	**<0.001**
I GCIPL vessel area density and ST VF CMS	0.683	**<0.001**
SN GCIPL vessel area density and IT VF CMS	0.462	**<0.001**
S GCIPL vessel area density and IT VF CMS	0.683	**<0.001**

VF = visual field, CMS = central mean sensitivity GCIPL = ganglion cell inner plexiform layer, SN = super-nasal, IN-infero-nasal, ST = supero-temporal, IT = infero-temporal, I = inferior, S = superior.

Sectoral analysis for the other OCTA parameters also showed statistically significant correlations between VSD and VPI in all sectors (p < 0.001), except for the ST GCIPL parameters and IN VF CMS (p = 0.097). Vessel diameter and VCI were found to be insignificantly correlated with VF CMS in all sectoral analysis parameters except for SN GCIPL and IT VF CMS (Table 5).

**Table 5:** Pearson correlation between sectoral OCTA parameters and sectoral visual field sensitivities for the glaucoma patients according to the structure-function correspondence map suggested by Garway-Heath et al. (N = 54).

	Vessel diameter		Vessel skeleton density		Vessel perimeter index		Vessel complexity index	
	Correlation	*P-*value	Correlation	*P-*value	Correlation	*P-*value	Correlation	*P-*value
IT GCIPL and SN VF CMS	-0.032	0.818	0.547	**<0.001**	0.544	**<0.001**	-0.267	0.051
ST GCIPL and IN VF CMS	-0.033	0.815	0.245	0.075	0.240	0.080	0.137	0.324
IN GCIPL and ST VF CMS	-0.081	0.561	0.560	**<0.001**	0.540	**<0.001**	-0.072	0.604
I GCIPL ST VF CMS	0.022	0.875	0.659	**<0.001**	0.669	**<0.001**	-0.246	0.073
SN GCIPL and IT VF CMS	0.072	0.603	0.481	**<0.001**	0.453	**<0.001**	-0.306	**0.024**
S GCIPL and IT VF CMS	0.007	0.958	0.655	**<0.001**	0.675	**<0.001**	-0.179	0.196

VF = visual field, CMS = central mean sensitivity GCIPL = ganglion cell inner plexiform layer, SN = super-nasal, IN-infero-nasal, ST = supero-temporal, IT = infero-temporal, I = inferior, S = superior.

**Fig 2:**
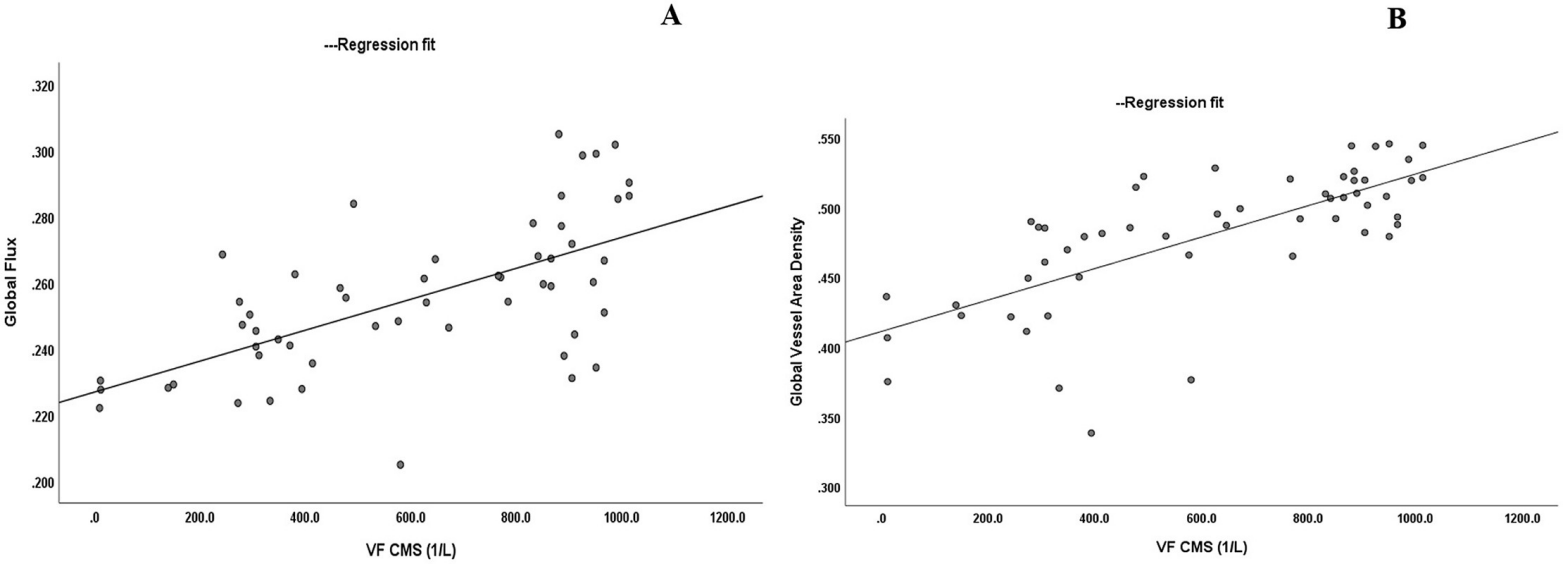
Scatter plots showing the correlations between the Visual Field Central Mean Sensitivity (VF CMS 1/L) and Ganglion Cell Inner Plexiform Layer OCTA global flux (A) and global vessel area density (B).

Fig 2 shows the linear relationship when VF CMS (anti-log CMS, 1/L) was compared to both GCIPL OCTA global flux and global VAD.

## Discussion

Evaluating the strength of structural and functional associations in glaucoma is essential to improve our ability to detect the presence and progression of glaucomatous damage and can have clinical implications, as the use of the stronger region could provide better detection and follow up in glaucoma for patients who may present with an early stage of macular VF defects. In the present study, we investigated macular vascular microcirculation in the GCIPL among eyes with open-angle glaucoma with different severity stages using OCTA and VF assessment methods. Global macular vascular microcirculation in the GCIPL, as determined by OCTA, was significantly correlated with global VF CMS in glaucomatous patients. Sectoral macular vascular microcirculation was also significantly correlated with sectoral VF CMS in all sectors except for the ST GCIPL and IN VF CMS. Other parameters such as vessel diameter and VCI were not significantly correlated with functional changes.

Shin et al. [5] studied the relationship between macular GCIPL and peripapillary RNFL measurements in glaucomatous eyes using HD-OCT and 24–2 VF sensitivities. They found statistically significant correlations between the corresponding VF sensitivity and the macular GCIPL thickness in all GCIPL sectors. Additionally, they reported that among six GCIPL sectors, the strongest association was observed between supero-nasal CMS and infero-temporal GCIPL thickness. The infero-temporal macula has been previously described as the macular vulnerability zone [23], and prior histologic studies in human [24, 25] and monkey [26] eyes have shown that in the central retina, there are more ganglion cells in the nasal and superior sectors than in the temporal and inferior sectors, respectively. In our study, the highest correlation was observed for both I GCIPL VAD and ST VF CMS, and S GCIPL VAD and IT VF CMS (r = 0.683, p<0.001). However, when we compared the correlation coefficients there was no significant difference except for the ST GCIPL, which was not significantly correlated to its correspondent VF CMS.

Prior studies have used 3 x 3mm OCTA scans to study the macular region in eyes with glaucoma. Yarmohammadi et al. [15] investigated macular circulation in glaucoma patients with single hemifield 24–2 VF defect using 3 x 3mm scans, and demonstrated a stronger association of visual function with both peripapillary and macular vessel density compared with structural measurements in similar regions of the affected hemifields of glaucoma eyes (p < 0.05). They hypothesized that ‘these results could reflect the existence of dysfunctional retinal ganglion cells with lower metabolic demands or vascular dropout, while these cells have not atrophied enough to be detected via imaging the structural tissue’. Penteado et al. [16] found a significant association between macular vascular density assessed by OCTA central VF sensitivities assessed with 10–2 VF, however, they did not evaluate different sectors in the macular region. Additionally, other studies have used 6 x 6 mm OCTA scans to study the macular region in POAG vs normal eyes [27, 28], but found weaker associations than our study, likely because global 24–2 VF data was used for analysis, rather than central 10 degree data as in our study.

We chose to study the sectoral OCTA parameters to better understand the relationship of blood flow and glaucoma damage. The sectoral VF CMS was adapted from the structure-function correspondence map suggested by Garway-Heath et al. [22] (Fig 1). Prior studies have investigated the sectoral correlation between peripapillary VD and VF loss using the Garway-Heath map and found strong correlation in the infero-temporal (IT), temporal, infero-nasal and supero-temporal (ST) sectors, similar to the correlation between RNFL thickness and VF loss [29, 30]. In our study, sectoral macular vascular microcirculation was significantly correlated with sectoral VF CMS in all sectors except for the inferonasal VF CMS with supero-temporal GCIPL blood flow parameters. The superior region of the macula (inferior VF) has been shown to be less affected by glaucoma [31]. Additionally, the RGCs of this less-affected region project to the temporal quadrant of the disc, which is a region less susceptible to glaucomatous damage. This difference in vulnerability might explain the lack of significant correlation found in our study, however, further research is warranted.

The structure–function relationship in glaucoma has been commonly reported using a simple linear regression model [32–34], but several studies have highlighted the second order polynomial regression model as the best fit model [35–37]. In our study, we initially used LOWESS regression to assess the relationships between OCTA blood flow metrics and VF CMS (1/L). The main advantage of this analysis is that it does not require the specification of a function to fit a model to all the data in the sample. Given that our LOWESS curve suggested a linear relationship between global GCIPL flux and central VF sensitivity (using non-logarithmical (1/L) scale), and an almost linear relationship between global GCIPL VAD and central VF sensitivity (data not shown), we chose to report the linear regression models in our results as a linear model.

Our study has some limitations. We obtained our VF CMS by averaging the 12 central points of the 24–2 HVF. Although 24 degree VF testing is routinely used in glaucoma patients, 10 degree VF testing could have increased the sensitivity to detect parafoveal VF defects [37]. Also, although we calculated the VF CMS based on the adapted structure-function correspondence map suggested by Garway-Heath et al. [22], our sectoral OCTA map is an approximation based on the OCT thickness map and each parafoveal OCTA sector may not perfectly match the 24–2 VF topographically due to individual differences in macular anatomy or due to RGC displacement [24, 38]. Further research and analysis of OCTA data is recommended to better help understand the macular structure-function relationship and to apply future results to both improve early detection and directed management of glaucoma.

In conclusion, we found significant correlations between global and sectoral macular vascular microcirculation in the GCIPL and global and sectoral VF CMS in glaucomatous patients. To the best of our knowledge, this study is the first to compare GCIPL structure and different VF deficits using scanning patterns determined by an OCTA device. These results show that OCTA may aid macular structure-function relationship studies in glaucoma patients, though further research is warranted.

## Supporting information

S1 File (XLSX)
